# Non-Neuronal Functions of the M_2_ Muscarinic Acetylcholine Receptor 

**DOI:** 10.3390/genes4020171

**Published:** 2013-04-02

**Authors:** Wymke Ockenga, Sina Kühne, Simone Bocksberger, Antje Banning, Ritva Tikkanen

**Affiliations:** Institute of Biochemistry, Medical Faculty, University of Giessen, Friedrichstrasse 24, 35392 Giessen, Germany; E-Mails: Wymke.Ockenga@biochemie.med.uni-giessen.de (W.O.); Sina.Kuehne@biochemie.med.uni-giessen.de (S.K.); Simonebocksberger@web.de (S.B.); Antje.Banning@biochemie.med.uni-giessen.de (A.B.)

**Keywords:** acetylcholine, G proteins, signal transduction, muscarinic receptors

## Abstract

Acetylcholine is an important neurotransmitter whose effects are mediated by two classes of receptors. The nicotinic acetylcholine receptors are ion channels, whereas the muscarinic receptors belong to the large family of G protein coupled seven transmembrane helix receptors. Beyond its function in neuronal systems, it has become evident that acetylcholine also plays an important role in non-neuronal cells such as epithelial and immune cells. Furthermore, many cell types in the periphery are capable of synthesizing acetylcholine and express at least some of the receptors. In this review, we summarize the non-neuronal functions of the muscarinic acetylcholine receptors, especially those of the M_2_ muscarinic receptor in epithelial cells. We will review the mechanisms of signaling by the M_2_ receptor but also the cellular trafficking and ARF6 mediated endocytosis of this receptor, which play an important role in the regulation of signaling events. In addition, we provide an overview of the M_2_ receptor in human pathological conditions such as autoimmune diseases and cancer.

## 1. Introduction to Acetylcholine and Its Receptors

In the early 1920s, Otto Loewi identified the neurotransmitter acetylcholine as a substance that displays an inhibitory effect on heart functions. Due to its apparent release by the vagus nerve, acetylcholine was named “Vagusstoff”. In the 1960s, this discovery led to the detection of the respective acetylcholine binding nicotinic and muscarinic receptors [1,2,3]. Besides their function in the central nervous system, acetylcholine receptors have been shown to display important functions in non-neuronal cells. In fact, many peripheral cells of our body are capable of synthesizing acetylcholine and express at least some of the receptors. The nicotinic acetylcholine receptors are ion channels. Represented by five subtypes, namely M_1_, M_2_, M_3_, M_4_ and M_5_, the muscarinic acetylcholine receptors (mAChRs) belong to the seven transmembrane helix receptor superfamily which activate trimeric guanine nucleotide binding proteins (G proteins) [4,5]. The genes encoding the five mAChRs are found in different genetic loci in humans: 11q12-13 (CHRM1), 7q35-36 (CHRM2), 1q43-44 (CHRM3), 11p12-p11.2 (CHRM4) and 15q26 (CHRM5). The mAChR genes are highly compact in that they do not contain any introns, and the resulting gene products are highly homologous among mammalian species [5,6].

In general, mAChR subtypes are differentially expressed in various cell lines and tissues, which all seem to express several receptor types. The expression pattern results in functional differences, which may sometimes be hard to assign to a specific receptor type. The non-neuronal functions of muscarinic receptors are summarized in Section 2.

On the basis of their signaling mechanism, mAChRs can be divided into two major classes [7]. The stimulation of M_1_, M_3_ and M_5_ receptor subtypes increases the intracellular calcium concentration upon binding of pertussis toxin insensitive G proteins (G_q/11_) and mediates the activation of the membrane bound enzyme phospholipase C (PLC) [8,9]. In contrast, M_2_ and M_4_ receptors mainly regulate the inhibition of adenylyl cyclase and the activation of potassium conductivity via pertussis toxin sensitive G proteins of the G_i_ and G_o_ classes. However, all mAChRs can in principle affect adenylyl cyclase activity and thus lead to an increase or decrease of cAMP by means of non-canonical signaling [10,11]. The mechanisms of muscarinic receptor signaling are more closely reviewed in Section 3.

In structural terms, the muscarinic receptors consist of seven relatively well conserved transmembrane domains (TM). These domains are connected through three extracellular loops, including the extracellular amino terminus, which exhibits a high variability among the five mAChR subtypes, and three intracellular loops. In addition to the TMs, it has been reported that the C-terminal tail of the M_2_ receptor is associated with membranes by palmitoylation [12]. The acetylcholine binding cavity is formed by the extracellular face of the TMs, with TM3 providing the bottom of the cavity, whereas TM2 and TM7 narrow it down. In an activated state, the mAChRs bind G proteins within their third intracellular loop and catalyze GDP-GTP exchange, leading to downstream signaling events [10,13]. In this review, we have focused on the muscarinic receptor subtype M_2_ whose structure, signaling and cellular trafficking are the subject of Section 4. In this section, we also summarize the importance of the small GTPase ARF6 for the endocytosis and trafficking of the M_2_ receptor and G protein coupled receptors (GPCRs) in general.

Muscarinic AChRs frequently are targets of autoantibodies, resulting in autoimmune diseases. Due to their function in muscle contraction, they have also been suggested to participate in the pathogenesis of e.g., lung diseases. Importantly, it is known that mAChR subtypes play a key role in various cancers. Section 5 provides a short summary of the role of muscarinic receptors, especially that of the M_2_ receptor, in human diseases. Concluding remarks and future directions are addressed in Section 6. 

## 2. Functions of Muscarinic Acetylcholine Receptors in Non-neuronal Cells

The functions of the different muscarinic receptor subtypes in the non-neuronal cholinergic system are diverse, depending on the distribution of single receptor subtypes in different tissues. Cells expressing mAChRs can be found virtually everywhere in the body. They are present e.g., in the epithelial layer of the airways, the skin, the immune system, the urinary bladder, reproductive organs, vascular endothelial cells, connective tissue, muscles and even tendons. Genetically ablated mice have been generated for all five muscarinic receptor subtypes [14,15,16,17,18]. These mice are viable and show no major developmental defects. However, some specific functions of receptor subtypes have been conclusively demonstrated in these mice (Summarized in [19]). In this paper, we are not aiming at providing an exhaustive review of the non-neuronal mAChR functions. For this, the reader should refer to recent excellent reviews in this topic [20,21,22]. 

Keratinocytes of the skin display the mRNA for all five muscarinic subtypes, and acetylcholine apparently plays a role in migration, differentiation, proliferation and adhesion of the cells [23,24,25]. In keratinocytes, it was shown that, in conjunction with nicotinic AChRs, muscarinic receptors are also necessary e.g., for the development of epithelial architecture and thus barrier formation. M_1_ and M_4_ receptors are well expressed in the suprabasal layer of the human skin, whereas M_2_, M_3_ and M_4_ receptors are found especially in the basal layer, as shown by *in situ* hybridization and antibody staining of skin sections [3,20]. As keratinocytes synthesize and secrete acetylcholine for autocrine stimulation, it is plausible that the secreted acetylcholine also affects melanocytes which express M_1_ through M_5_ receptors, detectable both at mRNA and protein level [26]. The migration of human keratinocytes is regulated in a reciprocal manner by two different muscarinic receptors. On the one hand, the M_4_ receptor induces migration and wound re-epithelialization, whereas M_3_ inhibits these processes. Accordingly, the M_4_ receptor upregulates the expression of the integrins α5β1, αVβ5, and αVβ6, which are connected to the movement of the cell, and the M_3_ receptor causes the expression of the integrins α2β1 and α3β1 that promote a sedentary state of the cell [27]. In fact, an acetylcholine gradient has even been shown to induce the migration of skin keratinocytes upwards in the epidermis [28].

The urothelium of the urinary bladder also expresses all of the muscarinic receptor subtypes of which M_2_ is the most prominently expressed one. Quantitative PCR and immunohistochemistry experiments showed that M_1_ and M_2_ are predominantly expressed by specific cell types in the urothelium. The M_1_ receptor is found in basal cells, and the M_2_ receptor shows a high expression in umbrella cells. M_3_ and M_4_ receptors are more homogenously distributed, and the M_5_ receptor content decreases from luminal to the basal cells [29]. Furthermore, the M_3_ receptor seems to be important for the contraction of the detrusor muscle [30] and generally appears to be the major regulator of smooth muscle contraction, as evidenced by knockout mouse studies [19]. 

In the epithelium of the airways, the M_1_ receptor plays a vital role. Together with nicotinic acetylcholine receptors, it stimulates the proliferation of the epithelial cells [31]. Moreover, ciliar activity, chloride secretion and thus the mucociliary clearance are mediated by muscarinic receptors [32]. Furthermore, M_3_ receptors stimulate while M_2_ receptors inhibit the cilia driven particle transport in the airways [33]. In addition to these receptor subtypes, the M_4_ receptor is highly abundant in peripheral lung tissues and has been suggested to play a role in autoinhibition of acetylcholine release in the trachea [34].

In regard to mediation of parasympathetic control, the M_2_ receptor is so far considered as the only muscarinic receptor subtype able to influence cardiac functions, e.g., heart rate or contractility. However, also M_1_ and M_3_ receptor proteins are expressed in the membrane of human cardiac cells [35] and participate in the control of the blood circulation system. Some major vasoactive mediators are nitric oxide, endothelium derived hyperpolarizing factor and prostanoids. All these substances are supplied by endothelial cells upon stimulation of their muscarinic receptors. Acetylcholine release itself might be regulated by the blood flow, shearing forces and blood pressure and may therefore modulate the release of the vasoactive mediators [22]. In line with this, release of acetylcholine has been shown as a consequence of increased blood flow in cultured human umbilical vein endothelial cells [36].

The non-neuronal cholinergic system also plays an important role in the immune system. The mRNAs for all five muscarinic receptors are present in most human mononuclear leukocytes, human leukemic cell lines and also in animal immune cells [20,37,38]. During immunological reactions, the autocrine and paracrine stimulation of macrophages, T cells or dendritic cells induces immune responses such as the expression of interleukin-2 and its receptor [8,38]. Depending on the immune status, the expression of muscarinic receptors varies in T cells. In non activated CD4 and CD8 cells, M_1_, M_3_, M_4_ and M_5_ receptors are present. This pattern changes upon activation of the cells via T cell receptor crosslinking, in that M_1_ and M_5_ receptors become upregulated in CD4 cells. In contrast, CD8 cells show an increased expression of M_1_ and M_4_ receptors, while the M_3_ receptor is downregulated [39]. Nevertheless, the M_5_ receptor seems to be the most important muscarinic receptor subtype in B and T cells, as the mRNA of this receptor subtype is upregulated upon activation in many of these cell types. The immunological activation therefore requires the cholinergic transmission for correct cell differentiation [40]. M_5_, together with M_1_ receptors, is also involved in modulation of the immune system as it is important for the regulation of cytokine production [41]. Another fundamental mechanism in the human body that is regulated by the non-neuronal cholinergic system is the release of insulin from β-pancreatic cells, which serves to maintain glucose homeostasis. It is mainly induced by glucose but at the same time, it can also be facilitated by muscarinic receptors. In this context, it is the M_3_ receptor delivering the stimulatory effects on the insulin release and potentiating it [42].

## 3. Signaling by the Muscarinic Acetylcholine Receptors

**Figure 1:**
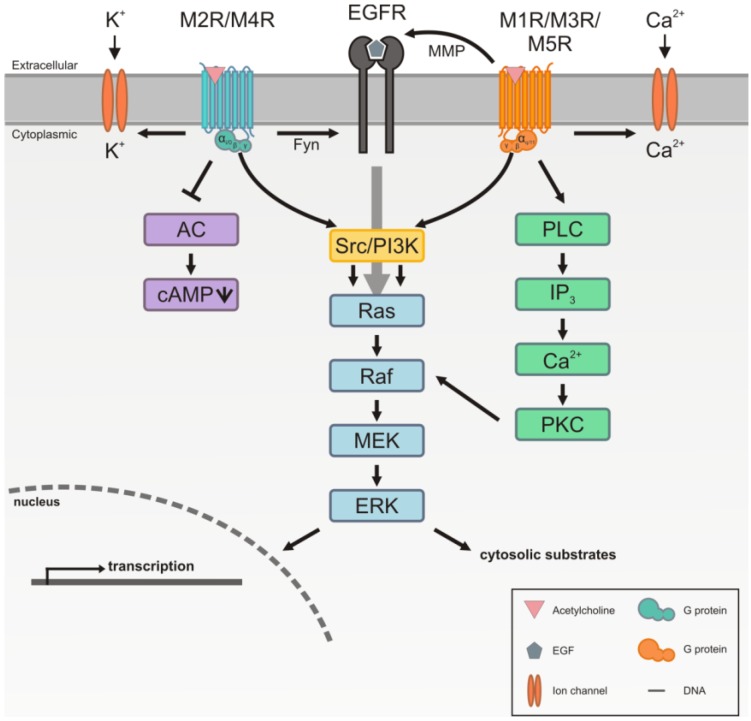
Canonical signaling of muscarinic receptors. The muscarinic acetylcholine receptors (mAChRs) are coupled to trimeric G proteins that consist of α-, β- and γ-subunits. M_2_ and M_4_ receptors couple preferentially to G_i/0_, whereas M_1_, M_3_ and M_5_ receptors mainly couple to G_q/11_. Upon stimulation with acetylcholine or a related agonist, M_2_ and M_4_ receptors inhibit the activity of the adenylyl cyclase (AC), leading to a decrease in intracellular cAMP levels. In addition, both receptor subgroups can activate ion channels. M_1_, M_3_ and M_5_ receptors activate PKC by means of upstream PLC activation and increase in IP_3_ and Ca^2+^ levels. PKC can activate Raf kinase, leading to the activation of the MAP kinase cascade and ERK1/2. Pathways that are common for all receptor subtypes are the activation of ERK1/2 via a Src/PI3K pathway or transactivation of the EGF receptor. Transactivation of EGFR by M_2_ and M_4_ receptors occurs in a Fyn dependent mechanism, whereas M_1_, M_3_ and M_5_ utilize matrix metalloproteases (MMP) that release extracellular, EGF like peptides.

The muscarinic acetylcholine receptors belong to the family of GPCRs, comprising seven transmembrane domains. They initiate the signal transduction through their coupling with heterotrimeric guanine nucleotide binding proteins, the so called trimeric G proteins. These G proteins consist of α-, β- and γ-subunits which couple to the muscarinic receptors mainly at the receptor’s third intracellular (i3) loop. However, the second intracellular loop and the C-terminus have also been suggested to be involved [43]. Upon binding of a ligand, GPCRs are conformationally rearranged. This results in an exchange of bound GDP to GTP in the α-subunit, which then leaves the complex, whereas the β- and γ-subunits stay together to facilitate signal transduction. There are several isoforms of the G proteins, and due to their preferential coupling to a different subset, muscarinic receptors can be divided into two distinct groups. The M_1_, M_3_ and M_5_ receptor subtypes preferentially couple to G_q/11_, whereas M_2_ and M_4_ receptors couple to G_i/o_. Depending on the class of the G protein involved, different downstream effectors are activated in a cell upon receptor stimulation. The typical signaling pathways of mAChRs are summarized in Figure 1. Through their linkage to G_q/11_, M_1_, M_3_ and M_5_ receptors predominantly activate PLC via the α-subunit. The activation of PLC results in production of diacylglycerol (DAG), which is generated together with inositol trisphosphate (IP_3_) upon the hydrolysis of phosphatidylinositol 4,5-bisphosphate (PIP_2_) by PLC. This path facilitates the mobilization of intracellular Ca^2+^ and activation of the protein kinase C (PKC). On the other hand, M_2_ and M_4_ receptors mainly inhibit the adenylyl cyclase through their corresponding G proteins, leading to a decrease in cAMP levels. However, this provides only a simplified view of the basal signaling events, and there are a lot more proteins involved in the fine tuning and regulation. In addition, the βγ-dimer is also able to participate in the signal transduction. 

In accordance with the fact that there are 27 α-, five β- and 14 γ-subunit isoforms in the human genome for the heterotrimeric G proteins [44], several different signaling pathways can be stimulated by the muscarinic receptors. For example, phospholipase D (PLD) can be activated via G_12_ coupled to M_3_ receptors [45], and M_2_ as well as M_3_ receptors can activate the sphingosine kinase to mobilize Ca^2+^ via sphingosine-1-phosphate [46]. Furthermore, the signaling can be influenced by the Regulator of G protein Signaling (RGS) proteins which can accelerate the inactivation of G proteins and thus reduce their signaling [47]. Muscarinic receptors are also able to act on different ion channels in the cell membrane. Whereas M_2_ and M_4_ receptors can directly activate inward rectifying potassium channels via their βγ-subunits [48], the M_1_ receptor can inhibit this conductance [49]. In addition, M_1_, M_3_ and M_5_ receptors have the ability to activate calcium channels, which has been shown to be dependent on G protein activation [50,51].

A large scale approach to identify interaction partners of muscarinic receptors was done by Borroto-Escuela *et al.* by means of a tandem affinity purification method and subsequent mass spectrometry [52,53]. A wide number of proteins were selectively recruited upon carbachol stimulation of a specific receptor. For the M_1_ receptor, these included PLC β1, G protein coupled kinases GRK2 and GRK6, as well as the focal adhesion kinase. Proteins that were detected together with M_1_ receptors even in unstimulated neuroblastoma cells were caveolin-1 and -2, β-tubulin and ADP ribosylation factor 6 (ARF6). The trimeric G proteins that were found to be receptor associated were the Gα isoforms q, 11, 12 and 13 for M_1_, M_3_ and M_5_ receptors, and Gα i1, i2 and i3 for the M_2_ and M_4_ receptors. Furthermore, association of Gβ isoforms 1 and 2 as well as Gγ isoforms 2, 7 and 11 was also identified.

A downstream signaling cascade that is accessed by all subtypes of muscarinic receptors is the mitogen activated protein (MAP) kinase pathway. For M_1_, M_3_ and M_5_ receptors, the activation of the MAP kinase pathway typically involves the activation of PKC. This cascade includes the Gα_q/11_ mediated activation of PLC and the resulting production of DAG and IP_3_, as described above. In human neuroblastoma SK-N-BE2(C) cells, the MAP kinase pathway proceeds via a sequential activation of Ras, Raf and MEK [54], whereas in the case of M_1_, M_3_ and M_5_ receptors, the involvement of Raf but not Ras is more typical [55,56]. Beside these, there are pathways leading to the phosphorylation and therefore activation of the extracellular signal regulated kinases ERK1/2, which are PKC independent. In some cases, the signaling is mediated by the G_βγ_ dimer and Src or phosphatidylinositol 3-kinases (PI3K) [57,58]. Other alternative pathways for these receptors to activate MAP kinases, in a PKC dependent or independent manner, involve a subset of G proteins different from G_q/11_ [59,60]. Even though all muscarinic receptor subtypes can accomplish the activation of ERK1/2, not all of them significantly contribute to ERK activation in a single cell type. In PC12D cells, M_1_, M_4_ and M_5_ receptor mRNAs are present but only the M_1_ receptor appears to contribute to the phosphorylation of ERK1/2, although M_1_ represents only a small proportion of the population of muscarinic receptors at the protein level in these cells [59]. M_2_ and M_4_ receptors preferentially utilize the βγ subunits of G_i_ for the signaling to the MAP kinases. In COS-7 cells, this pathway involves the recruitment of PI3Kγ to the plasma membrane and the activation of a tyrosine kinase, as well as Shc, Grb2, Sos, Ras and Raf, which at the end results in the phosphorylation of ERK1/2 [61]. 

Beside ERK1/2, the c-Jun N-terminal kinases (JNKs) are another group of MAP kinases that can be targets of muscarinic signaling. In COS-7 cells, they are brought into action via M_1_ and M_2_ receptors in a Ras and Rac1 dependent manner, induced by the βγ-subunits of the receptors [62]. However, JNKs can also be activated via the α-subunit of a GPCR, as is the case for the α-subunit of G_11_ coupled to the M_1_ receptor in human embryonic kidney (HEK)-293 cells [63]. The activation of JNKs can occur simultaneously with ERK1/2, as ERK1/2 and JNK are both activated by overexpressed M_3_ receptors in Chinese hamster ovary (CHO) cells. On the contrary, ligand binding to M_2_ receptors expressed at a comparable level only results in the phosphorylation of ERK1/2. Activation of ERK1/2 through these pathways has been shown to be independent of changes in the intracellular Ca^2+^ concentration [64]. Further members of the MAP kinase family, the p38 mitogen-activated protein kinases, can also be stimulated by the muscarinic receptors. The activation of p38 by M_1_ receptors involves the α- and βγ-subunits, whereas the M_2_ receptor only utilizes the βγ-subunits in HEK 293 cells [65]. This pathway is also dependent on PKC and Src kinase signaling [63]. 

After ligand binding and stimulation, muscarinic receptors are desensitized in a process that is dependent on receptor phosphorylation at serine and threonine residues by members of the GRK family [66]. These GRKs only work on agonist bound receptors, thus leading to a specific desensitization after stimulation. Phosphorylation of the receptor allows β-arrestins to bind, which suppresses the G protein interactions and therefore terminates the signal. In unstimulated cells, GRKs are located in the cytoplasm and only translocate to the receptors at the plasma membrane upon stimulation. They bind to the released βγ-subunits and PIP_2_ [67]. This desensitization process underlies a negative feedback loop, as ERK1/2 can phosphorylate GRK2 and decrease its activity [68,69]. Besides its role in desensitization, the phosphorylation of muscarinic receptors is also important for their internalization. Again, β-arrestins are involved in most cases, and their binding is necessary for an accurate endocytosis of the muscarinic receptors. However, the dominant negative β-arrestin mutant V35D only displayed a significant effect on M_1_, M_3_ and M_4_ receptor sequestration, indicating that the receptor subtypes may differ in their requirement for β-arrestin [70]. Upon internalization, the receptors can either be resensitized or degraded. Resensitization can be blocked by applying protein phosphatase inhibitors, likely through inhibition of receptor dephosphorylation in the endosomal compartments [71].

## 4. The M_2_ Muscarinic Acetylcholine Receptor

### 4.1. Structural Features of the M_2_ Receptor

The M_2_ receptor subtype is apparently different from all the other muscarinic receptors in several respects. Even though the M_2_ receptor is coupled to G_i/o_, similarly to M_4_ receptor, it exhibits some properties that are characteristic only for this receptor subtype. Whereas all other muscarinic receptors are resensitized and recycled back to the plasma membrane in different cell types, the M_2_ receptor is usually downregulated by degradation [72]. Furthermore, its internalization appears to differ in the requirement for β-arrestin and dynamin [70,73].

**Figure 2:**
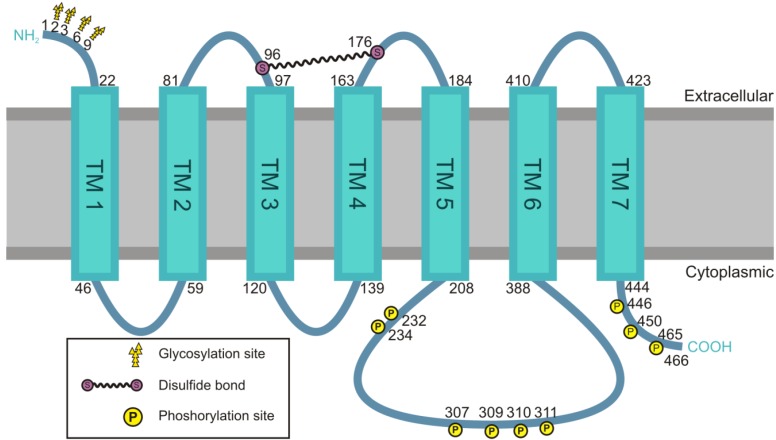
Structure of the M_2_ receptor. The M_2_ muscarinic acetylcholine receptor belongs to the family of G protein coupled receptors. The seven transmembrane domains (TM1-7) are connected by intracellular or extracellular loops, and the receptor exhibits four glycosylation sites at its extracellular N-terminal domain. The serine residues 96 and 176 in the first and second extracellular loop are connected to each other by a disulfide bond. Except for the third intracellular (i3) loop, other loops are relatively small. The i3 loop consists of 181 amino acids and has no specific secondary structure. The C-terminal domain and the i3 loop can be phosphorylated at several residues.

The most important structural part of the M_2_ receptor that facilitates the signaling process is, as with the other subtypes, the i3 loop of the receptor. The structure of the M_2_ receptor is depicted in Figure 2. A recent study has described the atomic structure of the M_2_ receptor, revealing interesting common features but also differences to other muscarinic receptors [74]. Unfortunately, this structure does not contain the i3 loop which turned out to be too flexible to allow for a structure determination at a sufficiently high resolution. The i3 loops of the muscarinic receptors are relatively long and consist of 160 to 240 amino acid residues. They represent the part of the receptor that is responsible for most of the differences between the single subtypes. The first and second intracellular loops show sequence similarities of approximately 90% between receptor subtypes, whereas the i3 loops merely feature 40% identity in their amino acid sequence, accompanied by a high variability in their length [52]. The i3 loop of the M_2_ receptor exhibits a flexible structure without specific secondary structure elements, except for the region at the C- and N-terminal part that may form α-helices [75]. A short sequence of four amino acids at the C-terminal end of the i3 loop, close to the sixth TM, mediates the coupling of the M_2_ receptor to the α-subunit of G_i/0_ [76]. The i3 loop is also the most important section where the βγ-subunits of the heterotrimeric G proteins bind. Furthermore, it is phosphorylated by GRK2 upon stimulation and plays an important role in receptor internalization [77,78,79]. 

The M_2_ receptor’s affinity for its ligand, the internalization and efficiency of signal transduction can be influenced by the cholesterol content of the plasma membrane, and depletion of cholesterol leads to a reduced ligand affinity, lower rates of receptor internalization but an increased inhibition of the adenylyl cyclase [80]. However, although this would suggest the involvement of the cholesterol rich, lipid raft like microdomains, M_2_ receptors are not localized in rafts at the plasma membrane of Madin-Darby Canine Kidney (MDCK) cells, and rafts are not involved in their apical targeting [81]. In polarized MDCK cells, the apical sorting signal for the M_2_ receptor was found to reside in the i3 loop [81]. However, the apical targeting of M_2_ receptors in polarized MDCK cells is independent of glycosylation, and the expression and correct function of the receptor do not require glycosylation [81,82].

It has become clear that the M_2_ receptor is not found as a monomer but that it homo-oligomerizes to a certain degree [83]. Using fluorescence resonance energy transfer approaches, the M_2_ receptor was identified to reside in the membrane of CHO cells as a tetramer [84]. Beyond that, the existence of hetero-oligomers was discussed, and analysis using bioluminescence resonance energy transfer could demonstrate the formation of such oligomers between M_1_, M_2_ and M_3_ receptors [85]. The interaction between M_2_ and M_3_ receptors is likely to be mediated by electrostatic interactions between the i3 loops [86]. Heterodimerization between M_2_ and M_3_ receptors has also been suggested by the data showing that coexpression of both receptors in JEG-3 cells results in increased lysosomal degradation of the M_3_ receptor which normally displays robust recycling rather than degradation [85]. This was attributed to a more dominant effect of the M_2_ receptor, which is predominantly sorted in the lysosomal pathway instead of being recycled. 

### 4.2. Signaling of the M_2_ Muscarinic Acetylcholine Receptor

As a G_i/o_ coupled receptor, the main effect of M_2_ receptor stimulation could be expected to be the inhibition of the adenylyl cyclase and a decrease in cellular cAMP levels. However, this appears not always to be the case. In CHO cells expressing M_2_ receptors, for example, also the net synthesis of cAMP can be stimulated upon agonist binding to the M_2_ receptor. In these cells, the M_2_ receptor also seems to couple to G_s_ or G_q/11_, thus changing the outcome of the receptor signaling [87].

Very important downstream targets of the M_2_ receptor signaling are the MAP kinases ERK1/2, which can be activated by different signaling pathways. In M_2_ receptor expressing COS-7 cells, the G protein βγ-subunits coupled to the M_2_ receptor are able to induce the phosphorylation of ERK1/2 via the PI3Kγ as a part of a pathway that involves a tyrosine kinase, Shc, Grb2, Sos, Ras and Raf [61], but the α-subunit can also be involved in ERK1/2 activation [88]. An isoform of Rap1 GTPase-activating protein, rap1GAPII, can bind specifically to the α-subunits of the G_i_ family. Upon stimulation of the M_2_ receptor, rap1GAPII decreases the amount of GTP bound Rap1. As active Rap1 antagonizes Ras function, the resulting inactivation of Rap1 represents a way of the α-subunit of the G_i_ coupled M_2_ receptor to facilitate ERK1/2 activation [88]. The transactivation of the epidermal growth factor receptor (EGFR) can also contribute to MAP kinase signaling following the stimulation of GPCRs. Upon extracellular ligand stimulus, EGFR dimerizes, its tyrosine kinase activity is promoted, and it becomes autophosphorylated. Thus, its downstream signaling pathways are activated and contribute to ERK1/2 phosphorylation. In fact, our unpublished data show that in HaCaT keratinocytes, most of the ERK activation upon carbachol stimulation can be attributed to EGFR transactivation. The transactivation has been shown in several cell lines [89], and it may involve the activation of matrix metalloproteases (MMP) that lead to the proteolytic release of EGF like peptides from the cell surface [90,91], as already demonstrated for M_1_ and M_3_ receptors [92,93]. However, for the M_2_ receptor an MMP independent mechanism dependent on the protein tyrosine kinase Fyn was established. In COS-7 cells transiently overexpressing M_2_ receptor, the M_2_ receptor mediated transactivation upon carbachol stimulation leads to an incomplete EGFR downstream signaling towards ERK1/2 and Akt, whereas PLCγ is not activated [94]. The authors postulated that differences in the tyrosine phosphorylation profiles of the EGFR might be generated depending on the activation mechanism, and the transactivation thus can result in an incomplete signaling. 

The interaction of β-arrestin with ERK1/2 is thought to inhibit its translocation to the nucleus, thus restricting the ERK signaling of GPCRs to the cytoplasm and changing the outcome of the ERK signaling [95,96]. Importantly, β-arrestins do not only inhibit the nuclear translocation but also function as scaffolds that bring together ERK2, MEK1 and Raf-1 [95]. An endosomal localization was observed for the overexpressed M_2_ receptor, ERK1/2 and β-arrestins in HeLa cells upon carbachol stimulation, whereas a more diffuse cytoplasmic distribution of β-arrestin was detected after stimulation upon overexpression of the other muscarinic receptors [97]. As the association of β-arrestin with the M_2_ receptor is stable only after stimulation, it is possible that the mechanisms of ERK1/2 activation differ to a great extent between the M_2_ receptor and the other muscarinic receptors. It can be hypothesized that the prolonged interaction of M_2_ receptor with β-arrestin might play a role to restrict the signal of this receptor subtype to the cytoplasm. Together with the G protein mediated ERK activation targeted to the nucleus, the β-arrestin mediated cytoplasmic signaling provides additional possibilities for the control of MAP kinase activity and substrates that become phosphorylated, applying a high level of complexity to the modulation of signaling. 

Upon agonist stimulation, the M_2_ receptor is rapidly phosphorylated on serine/threonine residues by second messenger kinases such as protein kinase A (PKA) or by specific G protein receptor kinases, e.g., GRK2 which appears to be the main kinase for the M_2_ receptor [98]. The targets of GRK2 are the serine and threonine residues in the central part of the i3 loop [99], and overexpression of a dominant negative GRK2 mutant together with the M_2_ receptor in HEK cells leads to a complete loss of M_2_ receptor desensitization [100]. Phosphorylation of the serines and threonines within the amino acid residues 307 to 311 upon receptor stimulation allows β-arrestin binding [101] which is required for the termination of M_2_ receptor signaling by disrupting the interaction of the receptor and its coupled G protein. It has been suggested that the phosphorylation dependent process of M_2_ receptor internalization is independent of β-arrestins and does not require clathrindependent endocytosis [101]. However, studies in mouse embryonic fibroblast derived from β-arrestin deficient mice showed that β-arrestin is indeed required for M_2_ receptor endocytosis [97]. The phosphorylation of M_2_ receptor in the i3 loop upon stimulation seems to act as a signal for desensitization and may help the β-arrestins to overcome a putative inhibitory signal that precludes inappropriate receptor binding [102]. Some aspartic and glutamic acid residues in close proximity to the phosphorylation sites are essential for an adequate response to receptor phosphorylation. However, they rather appear to be involved in β-arrestin binding than directly in phosphorylation, as mutating them into asparagines and glutamines had no effect on receptor phosphorylation but impaired β-arrestin binding [103].

### 4.3. Receptor Internalization and Its Connection to Signaling

**Figure 3:**
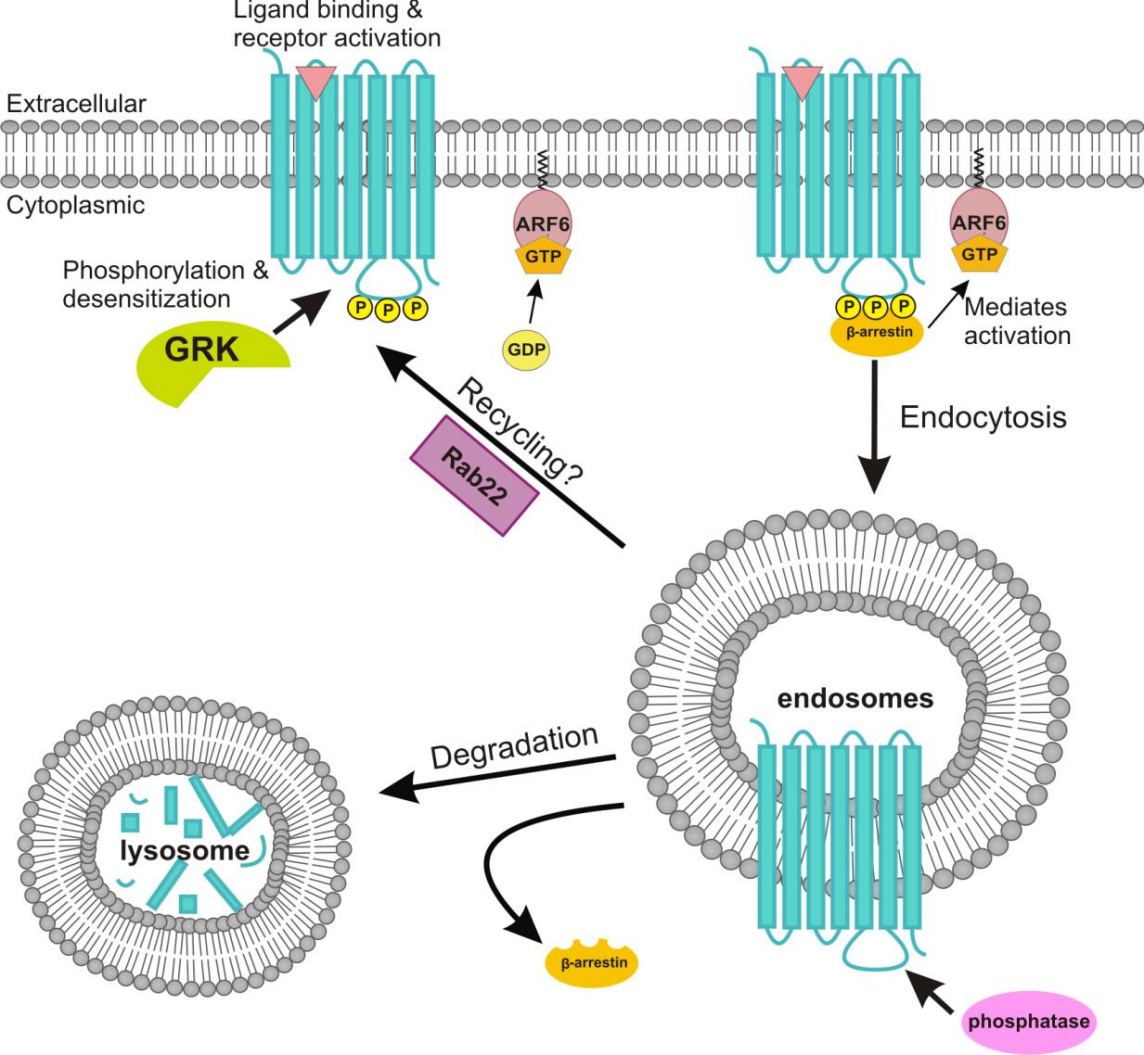
Agonist stimulated internalization of the M_2_ receptor. The M_2_ receptor is internalized by a clathrin-independent, ARF6-dependent pathway. Upon agonist stimulation, the M_2_ receptor is phosphorylated on serine/threonine residues by GRK, which facilitates the binding of β-arrestins and receptor desensitization. In addition, the β-arrestin activates ARF6, resulting in M_2_ receptor internalization in the early endosome. A phosphatase dephosphorylates the M_2_ receptor, causing β-arrestin dissociation from the receptor. The small G protein Rab22 has been implicated in the recycling of the M_2_ receptor. However, recycling appears to be a minor route for the M_2_ receptor which is mainly targeted to lysosomes and degraded.

Resensitization of GPCRs involves their dephosphorylation after endocytosis [104]. However, different from all the other muscarinic receptor subtypes, the M_2_ receptor is not resensitized in most cases and does not recycle back to the plasma membrane [72]. Figure 3 summarizes the main features of the M_2_ receptor endocytosis. While the internalization of the M_2_ receptor seems to be important for signal termination by means of receptor degradation, it does not seem to play a major role in the temporal regulation of the signaling. A comparative study of the extent and kinetics of receptor internalization for the different muscarinic receptor subtypes in CHO cells showed that the M_2_ receptor internalizes faster and more extensively than all the other receptors. Its degree of downregulation was comparable to those of M_3_, M_4_ and M_5_ receptors, and only the M_1_ receptor displayed a more intense degradation. The M_1_, M_3_ and M_5_ receptor downregulation exhibits a rapid component after a short term treatment with carbachol and a slower component for further downregulation upon long term agonist treatment, whereas this slower component was not apparent for the M_2_ and M_4_ receptors [105]. However, these results are not completely consistent with earlier studies. Roseberry and Hosey showed in HEK-293 cells that the ectopically expressed M_2_ receptor was rapidly and to a great extent internalized but only minimally degraded after 24 h of carbachol treatment. They even observed some degree of recycling of the receptor, albeit very slow, back to the plasma membrane [106]. Nevertheless, it is evident that there are differences in the mechanisms involved in receptor internalization and downregulation of the M_2_ receptor as compared to those of the other muscarinic receptors. However, special care needs to be taken when comparing studies addressing the trafficking and signaling of the receptors as these may be very much dependent on the cell type used. 

M_1_, M_3_ and M_4_ all undergo endocytosis in a clathrin and dynamin dependent manner. Even though it is evident that clathrin is not involved in M_2_ receptor endocytosis, the involvement of caveolin, β-arrestins and the GTPase dynamin in this process has remained less conclusive. Although an interaction between the M_2_ receptor and caveolin has been shown in cardiac myocytes [107], M_2_ receptor endocytosis takes place independently of caveolin in HEK-293 cells [108]. Endocytosis of many other GPCRs can be inhibited by means of expression of a dominant negative K44A mutant of dynamin2. Earlier studies concluded that dynamin would not be required for M_2_ receptor endocytosis [73], since the K44A dynamin2 mutant did not show any effect on M_2_ receptor endocytosis. More recent studies have used other mutants of dynamin2, such as the PIP_2_ independent K535M and a deletion mutant lacking the GTP binding domain. These studies could indeed show that dynamin is involved in M_2_ receptor endocytosis [109,110]. These discrepancies may be explained by unspecific effects of overexpression of the K44A and other dynamin mutants on cellular trafficking (Reviewed in [111]). Furthermore, it has recently been suggested that some dynamin functions may be independent of its GTPase activity, and thus different dynamin2 mutants may impair different steps during trafficking. Unfortunately, the dynamin dependency of M_2_ receptor trafficking has not yet been addressed using the specific chemical inhibitors of dynamin, such as Dynasore [112] that have recently become available.

The stimulation of M_1_ or M_2_ receptors leads to a stable ubiquitination of β-arrestin in mouse embryonic fibroblasts [113]. The internalization of the receptors is not affected by proteasome inhibitors but they completely block receptor downregulation which is increased upon expression of a constitutively ubiquitinated β-arrestin. However, the M_2_ receptor does not seem to undergo proteasomal degradation but is rather directed to lysosomes, indicating a role for β-arrestin ubiquitination in the correct lysosomal targeting of the receptor. Upon β-arrestin ubiquitination, the M_2_ receptor and β-arrestin stably colocalize in intracellular compartments, which was not observed for the M_1_ receptor [113]. It can be suggested that the pattern of ubiquitination on β-arrestin is important for the receptor targeting and the stable association of β-arrestin and the receptor. 

A protein connected to the M_2_ receptor whose role is not completely understood so far is the Receptor for Activated C Kinase 1 (RACK1), a protein enriched in M_2_ receptor immunoprecipitates, whose interaction with the receptor is disrupted upon treatment with the muscarinic agonist carbachol. RACK1 seems to prevent endocytosis, as a decreased expression of RACK1 increases the agonist mediated internalization of the receptor. However, at the same time, both overexpression and a decrease in RACK1 expression level inhibit receptor downregulation [114]. As RACK1 and β-arrestins have overlapping binding sites in the receptors [115], RACK1 might prevent β-arrestin binding to unstimulated receptors.

### 4.4. Role of ADP-Ribosylation Factor 6 in the Endocytosis of the M_2_ Receptor

The ADP ribosylation factor (ARF) family belongs to the Ras superfamily of GTP binding proteins which contains five major subclasses, namely Rab, Rho, Ras, Ran and ARF GTPases [116]. The six closely related mammalian ARF proteins are divided in three classes based on their sequence homologies. Accordingly, ARF1, ARF2 and ARF3 belong to class I and ARF4 and ARF5 to class II, whereas the sole member of class III is ARF6 [117,118]. As with other GTPases, ARF proteins switch between a GTP bound active and a GDP bound inactive form. The cycle of binding and hydrolysis of GTP is important for the localization of ARF proteins, and their activation is also connected with the membrane localization [118,119]. ARFs are able to interact with a plethora of regulatory proteins such as guanine nucleotide exchange factors (GEFs) and GTPase activating proteins (GAPs). Each ARF protein exhibits a preference for specialized GAPs and GEFs, and ARFs are specifically recruited to cellular compartments in which these regulatory proteins reside [119]. 

ARF6, together with ARF1, is the best studied family member of the ARF proteins, and its homologues have been found in many eukaryotes, including *Xenopus laevis* and *Drosophila melanogaster*, as well as in metazoans and fungi, whereas none appear to exist in plants. Similar to all members of the ARF family, ARF6 is myristoylated on the N-terminal glycine residue, which is important for the membrane association and thus influences the subcellular distribution of ARF6. This lipid modification is also essential for the activity of ARF6 in membrane trafficking [120,121,122,123] and for its membrane localization, as a non-myristoylated ARF6 (Gly2Ala mutation) was not able to bind to membranes and remained cytosolic [124]. 

ARF6 has been shown to be localized both at the plasma membrane and in endosomal compartments, and it evidently plays an important role in the regulation of peripheral membrane dynamics and the cortical actin cytoskeleton at the plasma membrane [117,125]. In line with this, ARF6 is a mediator of cytoskeletal rearrangements such as the formation of actin-rich plasma membrane protrusions [126,127]. ARF6 is also involved in Rac mediated membrane ruffling [126,127,128,129,130], phagocytosis [130] and regulation of secretion [131]. Furthermore, activated ARF6 has been indicated to promote the internalization of E-cadherin to early endosomes, inducing a disassembly of adherens junctions [132,133,134]. ARF6 mediated activation of PLD leads to the activation of phosphatidylinositol 4-phosphate 5-kinase (PIP5K), resulting in an increase of PIP_2_ at the cell periphery [135] where it is involved in clathrin dependent endocytic processes [136]. 

The requirement of ARF6 in membrane trafficking processes such as endosomal recycling is evidenced by the fact that the expression of a dominant negative ARF6 blocks recycling from endosomes. Thereafter, several studies have demonstrated the vital role of ARF6 in endosomal membrane trafficking [117,124,131,137,138]. Furthermore, ARF6 can regulate both the clathrin dependent as well as the clathrin independent endocytosis, but the ARF6 dependent endocytic pathway appears to be dynamin independent [133,139]. This is also suggested by the fact that depletion of ARF6 affects the endocytosis of several GPCRs, irrespective of their dependency on clathrin [140]. During clathrin independent endocytosis, activated ARF6 associates with the adaptor protein complex AP2 which mediates the post-endocytic sorting of ARF6 cargos, such as the major histocompatibility complex I (MHC1), β1-Integrin and the M_2_ receptor [141].

Stimulation of GPCRs not only activates downstream signaling cascades but also leads to internalization and desensitization of the respective receptor. In this process, the internalization is important for both the resensitization and degradation pathway [142,143,144]. It has been shown that ARF6 is important for the endocytosis of e.g., endothelin type B receptor, vasopressin type 2 receptor and the angiotensin type 1 receptor [140]. Stimulation of some GPCRs, such as β_2_-adrenergic and luteinizing receptors, results in the activation of ARF6, and overexpression of an ARF6 GAP reduces β_2_-adrenergic receptor internalization [145]. Furthermore, Claing *et al.* could show that β-arrestin regulates receptor endocytosis through the activation of and interaction with ARF6, thereby providing a link between the GPCR and endocytic proteins, such as clathrin and AP2 [146,147]. 

The M_2_ receptor has been demonstrated to be a cargo protein of ARF6 dependent endocytosis. Constitutively active ARF6 expression diminished the endocytosis but enhanced the rate and extent of recycling of the M_2_ receptor, implicating that the agonist stimulated internalization and perhaps also recycling of the M_2_ receptor is regulated by ARF6 [109,110,140]. However, a role for ARF6 in the ER-Golgi trafficking of GPCRs has also been suggested [148]. Reiner and Nathanson studied the involvement of several small G proteins in the regulation of the agonist induced endocytosis of the M_2_ receptor [109,110]. The dominant negative T22N mutant ARF6 and Rab22, a GTPase implicated in ARF dependent recycling [149], were able to substantially increase the M_2_ receptor endocytosis. M_2_ and M_4_ receptors initially utilize different endocytic pathways. Only later, they appear to end up in the same compartment which is positive for markers of clathrin dependent endocytosis [110]. Thus, the M_2_ receptor is initially internalized via a clathrin independent and ARF6 associated pathway, during which the M_2_ receptor is first observed in structures that lack the markers for clathrin dependent endocytosis. Thereafter, the M_2_ receptor internalization pathway quickly merges with the clathrin dependent pathway at the level of early endosomes, which contain the early endosome autoantigen 1 (EEA-1) [109]. 

## 5. Muscarinic Acetylcholine Receptors in Human Diseases

As with most GPCRs, muscarinic receptors are involved in human diseases by distinct mechanisms. In various autoimmune diseases, mAChRs frequently are targets of autoantibodies, which bind to the receptors and lead either to their activation or inactivation. Initially caused by the parasite *Trypanosoma cruzi*, the Chagas disease may lead to a heart failure and a sudden death upon disease progression. The involvement of the heart is possibly connected to the existence of autoantibodies in patients with Chagas disease against the M_2_ receptor, as these receptors display a negative chronotropic effect in cultured cardiomyocytes [150,151]. These autoantibodies against M_2_ receptor have been shown to induce receptor crosslinking and internalization due to activation of the receptor [152,153]. The Sjögren’s syndrome is a highly common autoimmune rheumatic disease, which is also connected to autoantibodies directed against the muscarinic receptors, among other antigens. Upon progression of the disease, lymphocytes infiltrate and attack the lacrimal and salivary glands, leading to dryness of the mouth and the eyes [154]. Autoantibodies recognizing the M_3_ receptor have been shown to be involved, leading to its inhibition and internalization. Due to the reduced signaling and impairment of secretion, the salivary flow rate is reduced [155,156]. In line with this, knockout mouse studies have shown that M_3_ receptor, together with M_1_ and M_4_ receptors, plays a major role in regulating salivary excretion [19]. 

Not only autoimmune diseases are related to the muscarinic receptors. In the Achilles tendon from patients with tendinosis exhibiting hypercellularity and hypervascularity, a higher level of M_2_ receptor can be observed in the endothelial cells of the vessels as compared to the tendons of healthy patients, suggesting the occurrence of acetylcholine and M_2_ receptor mediated vasodilatory mechanisms in the Achilles tendon [157]. Both M_2_ and M_3_ receptors affect the bronchial and tracheal smooth muscle contraction and are therefore likely to play a role in asthma and chronic obstructive pulmonary disease that are accompanied by an increased smooth muscle tone [158].

The expression of muscarinic receptors is described for a variety of cancer cells in which they appear to be highly involved in proliferation and migration. In the breast cancer cell line MCF-7, stimulation of muscarinic receptors leads to an activation of the ERK pathway in a manner dependent on PKC-ζ, phosphoinositide 3-kinase and a kinase of the Src family, increasing protein biosynthesis and cell proliferation [159]. A stimulatory effect on cell migration was shown for HeLa cells in an ERK1/2 dependent mechanism [160]. In addition, muscarinic receptors are thought to play a role in angiogenesis in breast cancer [161]. A cancer type that overexpresses muscarinic receptors is colon cancer, in which high levels of M_3_ receptors have been found to mediate cancer cell proliferation [162], and the M_3_ receptor is involved in cell migration of these cells via the release of MMPs and transactivation of EGF receptors [163,164]. This is clearly different from the influence of muscarinic receptors on the migration of keratinocytes, where the M_3_ receptor is rather inhibiting cell migration than promoting it [27]. 

Whereas M_3_ receptors stimulate cell proliferation of tumor cells, M_2_ receptors seem to have a contrary effect. A specific cancer type that is influenced by this muscarinic receptor is glioblastoma, a common brain tumor. In glioblastoma cell lines, addition of the M_2_ receptor agonist arecaidine decreased cell growth in a time and dose dependent manner and was counteracted by the M_2_ receptor antagonist gallamine. In contrast, muscarine had no significant influence on proliferation [165]. The effect of cholinergic ligands on small cell lung carcinoma cells is more controversial. On the one hand, cholinergic stimulation of the small cell lung cancer cells H69 enhances cell proliferation [166], but on the other hand, the SCC-9 cell line is inhibited in cell cycle progression upon cholinergic stimulation and shows a diminished DNA synthesis [167]. Another cell type that uses acetylcholine as a growth factor is hepatocellular carcinoma. In cancer tissues, the acetylcholinesterase is often downregulated and its decrease correlates with tumor aggressiveness [168]. Due to the diminished expression of acetylcholinesterase, acetylcholine cannot be hydrolyzed. It is likely that the elevated level of acetylcholine acts on muscarinic receptors of the tumor cells and therefore increases their proliferation. The above observations reveal how different the influence of muscarinic receptors on specific cells is, depending on the cellular environment and the cellular proteome. However, although muscarinic receptors sometimes exhibit unpredictable or unexpected effects on cancer progression, they still might be an interesting target for therapeutic drugs in the future.

## 6. Conclusions and Future Challenges

In tissues, the effects of acetylcholine are mediated by the repertoire of the receptor subtypes expressed, and both nicotinic and muscarinic receptors contribute to the cellular response observed. Since a certain cell type normally expresses more than one type of acetylcholine receptor, the cellular response is often a compiled one, with a contribution of several subtypes. Furthermore, the observed effects on the cell physiology are also dependent on the class of the G protein subunits and their modulators expressed by the specific cell type. Thus, the interpretation of the data resulting from studies in various cell types may be a complicated task. Some specific physiological functions of muscarinic receptor subtypes have been revealed by the studies on knockout mouse models (Reviewed in [169]). However, even in this system, one needs to be cautious since loss of one subtype may result in aberrant desensitization of another one due to a hypercholinergic status produced by the lack of the major receptor subtype in a certain tissue [170].

Pharmacological substances that result in the activation of only either of the receptor groups are available. However, the activation or even inhibition of a specific receptor subtype is notoriously difficult, as the substances used are rarely fully specific for a single receptor subtype. With increasing knowledge about the structural differences of the receptors, it may become possible to develop novel compounds that specifically affect the functions of a single subtype. Thereby, special interest has been paid on ligands that bind to allosteric regulatory sites. Intriguingly, the structure of the M_2_ receptor was recently solved [74] and revealed some important structural features specific for this subtype that may facilitate the generation of more selective drugs in the future. 

Older studies have frequently been performed using overexpression of a specific receptor subtype. However, if the receptor expression level is too high, this may even result in saturation of signaling or trafficking partners (“dominant negative effect”) and thus misinterpretation of the results. Furthermore, other receptors expressed in the cell type still contribute to the signaling response although the overexpressed one may be predominant. The discovery of specific gene knockdown strategies in cell culture, e.g., RNA interference, has provided better options to modulate the signaling in the target cells by depleting a specific receptor subtype. However, even this is not completely secure, as the other receptor subtypes expressed may compensate for the loss of another one. Interestingly, mouse embryonic fibroblast have been described not to contain mRNA or protein for any of the muscarinic receptors [97]. Thus, this cell type may be especially suitable for studies addressing the subtype specific signaling, provided that the required partners are also expressed.

Future challenges in the studies of non-neuronal functions of muscarinic receptors will certainly include revisiting the data obtained in various cell systems and by means of receptor overexpression. It will be of special importance to compile these data in a model that needs to be tested in a single system that allows definite conclusions on the function of a single receptor type. Special effort should also be dedicated to novel pharmaceutical compounds, both inhibitors and activators, which affect the function of only a specific muscarinic receptor. With these tools at hand, it will be possible to obtain a more conclusive picture about the non-neuronal functions of this receptor class. 
